# Mitochondrial DNA Analyses Indicate High Diversity, Expansive Population Growth and High Genetic Connectivity of Vent Copepods (Dirivultidae) across Different Oceans

**DOI:** 10.1371/journal.pone.0163776

**Published:** 2016-10-12

**Authors:** Sabine Gollner, Heiko Stuckas, Terue C. Kihara, Stefan Laurent, Sahar Kodami, Pedro Martinez Arbizu

**Affiliations:** 1 German Center for Marine Biodiversity Research (DZMB), Senckenberg am Meer, Wilhelmshaven, Germany; 2 Royal Netherlands Institute for Sea Research (NIOZ), Ocean Systems Sciences (OCS), 't Horntje, Texel, The Netherlands; 3 Senckenberg Natural History Collections Dresden, Museum of Zoology, Dresden, Germany; 4 School of Life Sciences, École Polytechnique Fédérale de Lausanne (EPFL), Lausanne, Switzerland; 5 Swiss Institute of Bioinformatics (SIB), Lausanne, Switzerland; UPMC, FRANCE

## Abstract

Communities in spatially fragmented deep-sea hydrothermal vents rich in polymetallic sulfides could soon face major disturbance events due to deep-sea mineral mining, such that unraveling patterns of gene flow between hydrothermal vent populations will be an important step in the development of conservation policies. Indeed, the time required by deep-sea populations to recover following habitat perturbations depends both on the direction of gene flow and the number of migrants available for re-colonization after disturbance. In this study we compare nine dirivultid copepod species across various geological settings. We analyze partial nucleotide sequences of the mtCOI gene and use divergence estimates (*F*_*ST*_) and haplotype networks to infer intraspecific population connectivity between vent sites. Furthermore, we evaluate contrasting scenarios of demographic population expansion/decline versus constant population size (using, for example, Tajima’s D). Our results indicate high diversity, population expansion and high connectivity of all copepod populations in all oceans. For example, haplotype diversity values range from 0.89 to 1 and F_ST_ values range from 0.001 to 0.11 for *Stygiopontius* species from the Central Indian Ridge, Mid Atlantic Ridge, East Pacific Rise, and Eastern Lau Spreading Center. We suggest that great abundance and high site occupancy by these species favor high genetic diversity. Two scenarios both showed similarly high connectivity: fast spreading centers with little distance between vent fields and slow spreading centers with greater distance between fields. This unexpected result may be due to some distinct frequency of natural disturbance events, or to aspects of individual life histories that affect realized rates of dispersal. However, our statistical performance analyses showed that at least 100 genomic regions should be sequenced to ensure accurate estimates of migration rate. Our demography parameters demonstrate that dirivultid populations are generally large and continuously undergoing population growth. Benthic and pelagic species abundance data support these findings.

## Introduction

Deep-sea hydrothermal vents are island habitats that occur globally along mid-ocean ridges, back-arc basins and island arcs. Tectonic events and volcanic eruptions make them unstable in space and time. The emergence of hot, sulfide- and mineral-rich hydrothermal fluids also characterizes this extreme ecosystem. Most vent macrofauna and several meiofauna species are restricted to the vent environment, where the sulfide-rich fluids nourish chemolithoautotrophic bacteria [1–3]. These bacteria form, as free-living communities or in symbiosis with macrofauna, the basis of the food-chain of a highly specialized, abundant, biomass rich but species poor vent community [4].

The rich sulfide mineral deposits at deep-sea hydrothermal vents could make this unique ecosystem a target area for the mining industry in the near future [5]. Seabed crawlers utilized in this industry use cutters to shred mineral deposits, resulting in large scale disturbances on the faunal communities living at hydrothermal vents [6]. Direct impacts include killing of fauna as well as removal of substrate and associated habitat modification (i.e. topography) and fragmentation [7, 8]. Conservation policies depend on predictions as to whether species have the potential to recolonize impacted areas and, hence, contribute to the recovery of communities after such major disturbance events.

Genetic analyses of so far undisturbed populations can greatly enhance our understanding of vent species population biology, and can allow predictions of recolonization and recovery. Recolonization potential can be described using the conceptual framework of population connectivity, a concept that describes the extent to what subpopulations exchange migrants. While a direct assessment of migration rates requires model-based demographic analyses of population genomic datasets [9–11], divergence indices (e.g. *F*_*ST*_ values) can provide indirect estimates of the extent to which the evolution of subpopulations is influenced by migration (genetic connectivity) [12]. In the context of conservation policies, genetic connectivity estimates can be complemented by assessments of genetic diversity and demographic characteristics. The combination of these different population genetic estimates allows characterization of putatively connected subpopulations with respect to their population size, their potential for adaptation under changing environmental conditions [13] and their propensity for expansive/restrictive population growth.

Genetic analyses have been previously applied for various macrofaunal and megafaunal species representing different superordinate taxa (e.g. Crustacea, Bivalvia, Gastropoda, Polychaeta) in distinct geographical settings, including the Mid Atlantic Ridge, East Pacific Rise, Eastern Lau Spreading Centre, and Central Indian Ridge [14]. These studies indicated that migration capability and gene flow between subpopulations of species is high [15–17]. However, though there are also some examples of pronounced genetic differentiation between subpopulations resulting from restricted gene flow, these examples often occur between geographically separated vent areas such as the Northern and Southern East Pacific Rise [18, 19]. In addition, there is also a general pattern of high intrapopulation genetic diversity and expansive population growth among various species from different vent sites [16, 20].

The underlying mechanisms of high connectivity (migration capability), high genetic diversity and expansive population growth across different species and taxa are not fully understood. On one hand, life history traits must play an important role, since they allow for rapid population growth and/or guarantee long-term planktonic larval duration as connectivity between invertebrate populations is often achieved by passive larval drift. On the other hand, passive larval drift is influenced by abiotic characteristics such as plume height or ocean currents [21]. Furthermore, the rate of exchange of individuals between vent fields is also likely influenced by vent field frequency and thus distance between single vent sites. Finally, major natural disturbance events such as volcanic eruptions can totally wipe out local populations [22–24], affecting population size and in turn exchange of individuals among populations [25].

Volcanic eruption and vent field frequency differ among hydrothermal vents at distinct spreading centers along mid-ocean ridges, back-arc basins and at volcanic arcs. Vent field frequency increases with increased spreading rate [26]. At the fast-spreading Eastern Lau Spreading Center (ELSC), the rate of spreading is 64 mm/yr and vent field frequency is ~8 per 100 km. On fast-spreading mid-ocean ridge at 9°N East Pacific Rise (EPR), the spreading rate is 98 mm/yr and vent field frequency is about 4 per 100 km. In contrast, on the slow-spreading Central Indian Ridge (CIR) (e.g., 42 mm/yr at 25°S) and on the Mid-Atlantic Ridge (MAR) (e.g., 23 mm/yr at 27°N), less than one vent field is present per 100 km [26]. The steady state assumption suggests frequent eruption intervals of ~10 years for fast-spreading ridges, and infrequent eruption intervals of ~1000 years or more for slow-spreading ridges [27]. As an example, at the 9°N EPR, two major volcanic eruptions have been documented in the last three decades—one in 1991 and the next in 2005/06 [28, 29]. Both eruptions killed the vast majority of animals in this area [22, 23, 30].

Disentangling to what extent geographic and geologic characteristics of vent fields (e.g., vent distance or frequency of volcanic eruption) can shape population genetic structure of invertebrate species, such as diversity, demography, connectivity, is indeed important for conservation plans; however, this remains challenging, not in the least because endemicity level per biogeographic province among vent macrofauna species is typically high (95%) and no vent species occurs circumglobally [31]. If such circumglobally existing species where available for investigation, comparative analyses could reveal how different geological and geographical vent field characteristics shape population structure under given species-specific life history traits. However, this concept further assumes comparable population histories in each biogeographic province.

In the absence of a circumglobally occurring model species that allow analyses of population structure at different vent regions, this study instead comparatively explores patterns of genetic diversity, demography, and divergence in populations of very closely related copepod species with similar life history traits across different oceans. We investigate nine species belonging to two genera within the vent endemic family Dirivultidae, a taxon with more than 50 described species. Dirivultidae is the one of the most species-rich invertebrate family at hydrothermal vents [32]. They are an important part of all vent communities and in the most extreme habitats, such as sulfide chimneys colonized by Pompeii worms (*Alvinella pompejana*), they are the most abundant animals. They are typically free-living among aggregations of foundation species such as Pompeii worms, tubeworms, snails, or bivalves [33, 34]. Some genera such as *Stygiopontius* and *Aphotopontius* occur at vents worldwide [35]; they typically have 4 eggs from which lecitotrophic nauplii hatch [36, 37]. Nauplii and copepodites have been observed in the pelagial above vents and also on the benthos [22, 38].

We have selected 9 dirivultid species from two genera from four areas: fast-spreading Eastern Lau Spreading Center (ELSC) and East Pacific Rise (9°N EPR), and slow-spreading Mid Atlantic Ridge (MAR 23°N, 26°N, 4°S), and Central Indian Ridge (CIR). We morphologically identify the species and use partial nucleotide sequences of the mitochondrial gene Cytochrome Oxidase I (mtCOI; ~650 bp) to estimate genetic diversity and demographic characteristics within populations. In addition, divergence between subpopulations is assessed to provide an estimate of genetic connectivity, i.e., describing to what extent gene flow affects evolutionary processes within populations. We further link genetic data to species abundance data in the benthos and in the pelagial, to the end of discussing our genetic results in the light of actual species population size and dispersal potential. Our analyses provide a basis for discussing whether the frequencies of vent fields and volcanic eruptions can shape the genetic composition of populations. Furthermore, based on observed population genetic patterns in dirivultid species in different oceans, we carry out statistical performance analyses and lay out a conceptual basis for future population genomic surveys in these populations. Finally, we use our data to estimate potential mining impact on this faunal group.

## Material and Methods

### Study areas, sampling, and sample processing

**Fig 1:**
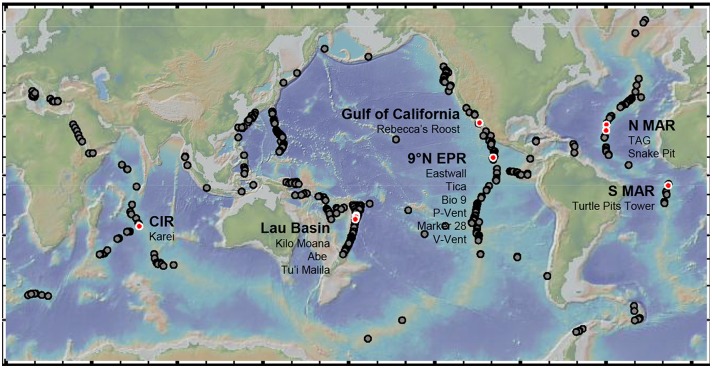
World map showing as grey circles vent sites under investigation during the last decades and as red circles vent sites we considered for this study. Name of studied biogeographic regions (in bold) and vent sites (listed from North to South) are given (CIR—Central Indian Ridge, EPR—East Pacfic Rise, MAR—Mid Atlantic Ridge).

Copepod specimens were collected by the submersible *Alvin* on the East Pacific Rise (EPR) and the Guaymas Basin (GB) in the East Pacific, by the submersible *Nautil*e on the Mid-Atlantic Ridge (MAR) in the Atlantic, by the ROV Jason the Eastern Lau Spreading Center (ELSC) in the West Pacific, and by the ROV Kiel 6000 on the Central Indian Ridge (CIR) in the Indian Ocean (Fig 1). All samples were taken in international waters, and did not contain any endangered species.

A map of the sites was created using GeoMapApp (http://www.geomapapp.org; version 3.6.2) and the interactive map from the interridge data base (http://vents-data.interridge.org/maps). Information on dive number, longitude, latitude and depth of sites where specimens were collected, as well as inter-site distances, is provided in Table 1.

**Table 1:** Dive number, details on study sites (biogeographical region, latitude (lat), longitude (long), depth (in m), site name, inter-site distances (dist.), and number of analyzed individuals per copepod species.

Dive	region	lat	long	depth (m)	site	dist. (km)	*S*. *h*.	*S*. *l*.	*S*. *b*.	*S*. sp. nov. 1	*S*. sp. nov. 2	*S*. *p*.	*A*. *l*.	*A*. *m*.	*A*. sp. nov. 1
AD 4457	GC	27°00'N	111°24'W	2000	Rebecca's Roost		1								
AD 4580	EPR	9°51'N	104°18W	2500	Eastwall	1903,3							4	4	
AD 4261 & AD 4580	EPR	9°50'N	104°17'W	2509	Tica	0,3	7							11 (10)	
AD 4266	EPR	9°50'N	104°17'W	2508	Bio 9	0,2	34 (32)								
AD 4575	EPR	9°50'N	104°17'W	2508	P-Vent	0,1							1		
AD 4465	EPR	9°50'N	104°17'W	2507	Marker 28	0,2	16								
AD 4368	EPR	9°47'N	104°17'W	2509	V-Vent	5,5	27								
J2#424 & J2#433	ELSC	20°03'S	176°08'W	2614	Kilo Moana			7	8						
J2#425 & J2#426	ELSC	20°46'S	177°11'W	2145	ABE	78,8		50	1						
J2#428 & J2#430	ELSC	21°59'S	176°34'W	1885	Tu'i Malila	141,2		24 (23)	26						
BICOSE—12–575	MAR	26°07'N	44°49'W	3620	TAG							19 (18)			
BICOSE—05–568	MAR	23°22'N	44°57'W	3450	Snake Pit	306,6						17 (15)			
M64/1	MAR	4°48'S	12°21'W	2992	Turtle Pits Tower	6890,8						2			
INDEX13—31ROV	CIR	25°19'S	70°02'E	2527	Karei										1
INDEX13—35ROV	CIR	25°19'S	70°02'E	2379	Karei					17	4				5

In case some of the gained sequences were too short or had too many ambiguities to apply population genetics, number of sequences used for population genetics is given in brackets. Sites are listed from North to South, and inter-site distance to next site is given in km. Region: EPR—East Pacific Rise, GC—Gulf of California (Guaymas Basin), ELSC—Eastern Lau Spreading Center (Lau Basin), MAR—Mid Atlantic Ridge, CIR—Central Indian Ridge; Species: S. h.–*Stygiopontius hispidulus*, S. l–*S*. *lauensis*, S. b.–*S*. *brevispina*, *S*. sp. nov. 1, *S*. sp. nov. 2, S. p.–*S*. *pectinatus*. 1, A. l.–*Aphotopontius limatulus*, A. m.–*A*. *mammillatus*, *A*. sp. nov. 1.

To obtain copepods, entire faunal aggregations were collected using different instruments such as mussel pots, slurp guns, or grabs by the submersibles’ or robots’ arms. After collection, faunal aggregations were put into isolated plastic boxes on the submersible/ROV and transported to the research vessel on the surface. Onboard the ships, samples were sieved over a 32 micrometer and 1 mm net successively to separate the meio- from the macrofauna. Live observation of copepods and other meiofauna under a dissecting microscope onboard the ship revealed that most animals collected were still alive. Meiofauna were immediately fixed in 99.5% EtOH. Back in the lab, meiofauna were sorted under a dissecting microscope to isolate the dirivultid copepods.

In this study, we morphologically identified and produced new sequences from the following species: *Stygiopontius* sp. nov. 1, *S*. sp. nov. 2, and *Aphotopontius* sp. nov. 1 from CIR, *S*. *pectinatus* from MAR, and *A*. *limatulus* and *A*. *mammillatus* from EPR (total 85 sequences; Table 1, S1 Table). In addition, we also include our priorly published mtCOI sequences for *S*. *lauensis* and *S*. *brevispina* (all ELSC), and for *S*. *hispidulus* (EPR) from Gollner et al. (2011) [39] (total 201 sequences; Table 1, S1 Table). Sample processing, morphological taxonomy, DNA isolation, PCR and sequencing were the same for the new and the previously-published specimens.

### Morphological Taxonomy

**Fig 2:**
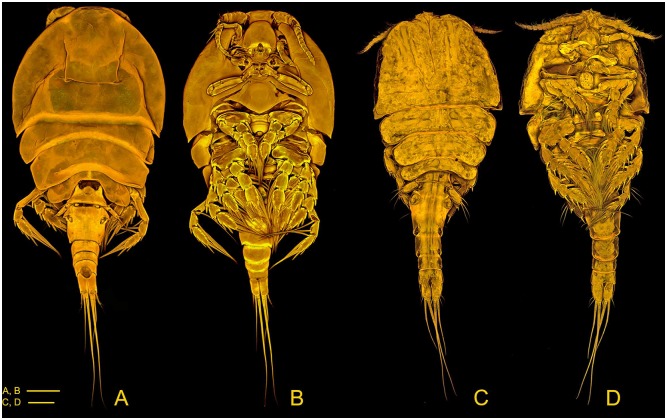
Confocal laser scanning microscopy of dirivulitd copepods. Maximum projections. *Aphotopontius* sp. nov. 1 Female A. habitus, dorsal; B. habitus, ventral. *Stygiopontius* sp. nov. 1 Female C. habitus, dorsal; D. habitus, ventral. Scale bars = 100 μm.

All specimens were morphologically identified to species level using the original species descriptions and a key to identify dirivultid copepod species [35]. Single individuals were put on glass slides in a small chamber filled with 99.5% EtOH and covered with a cover glass to reduce evaporation. In total, we used 286 specimens from 9 species in our study (Table 1). Three of the nine species are new to science: *Stygiopontius* sp. nov. 1, *S*. sp. nov. 2 and *Aphotopontius* sp. nov. 1 from the CIR (Fig 2). The new species will be morphologically described by Terue C. Kihara (in. prep.) and are presented in the Catalogue of INDEX [40]

### DNA isolation, PCR, and sequencing

The same animals that were morphologically identified were used for our mtCOI study. We applied the same methods for all specimens, published sequences and newly analyzed specimens alike. DNA was extracted from single specimens in 40 microliters of chelex (InstaGene Matrix, Bio-Rad) or 100 microl DNeasy Tissue Extraction Kit (QIAGEN, Hilden, Germany). We discovered no difference in PCR amplification success rate between these two extraction methods. Partial cytrochrome c oxidase subunit I (mtCOI) was PCR-amplified using universal primers LCOI (50-GGT CAA CAA ATC ATA AAG ATA TTG G-30) and HCOI (50-TAA ACT TCA GGG TGA CCA AAA AAT CA-30) [41] and GE Healthcare Illustra Pure Taq PCR beads. The mix for one individual consisted of 0.5 microl LCOI, 0.5 microl HCOI, 20 microl distilled H2O, and 4 microl of the DNA extract. Cycle conditions were 95°C for 5 min, 40 cycles at 95°C for 30 seconds, 42°C for 1 min, and 72°C for 1 min, and a final extension step at 72°C for 7 min. DNA was sent to Macrogen for Sanger-sequencing. Newly generated COI-sequences were manually quality controlled and clipped using BioEdit [42] and Chromas v2.23 (available at http://www.technelysium.com.au). Contaminations were extracted using BLAST against a non-copepod COI sequence database. Contig assembly of forward and reverse reads was completed using the Cap-Contig assembly function in BioEdit. All GenBank accession numbers, including newly generated and published ones from Gollner et al. [39] are given in S1 Table.

### Molecular taxonomy

The dataset used was a combination of the newly produced sequences and those from Gollner et al. 2011 [39]. Species specific codon-based nucleotide alignments for mtCOI were created using the software TranslatorX [43]. We used muscle [44] as an alignment algorithm, the translation table for invertebrate mitochondria, and allowed reading frame identification for each nucleotide sequence separately. This approach allows selecting sequences with protein coding open reading frames and excluding those with frame shift mutations or unexpected stop codons, i.e., sequences that may arise from nuclear mitochondrial insertions (NUMT). Species specific alignments where combined and subsequently used for phylogenetic inference with software RAxML (Maximum-likelihood method, 1000 fast bootstrap repeats, GTR-GAMMA as evolutionary model). Species from harpacticoid copepods were used as outgroup (*Ameira* species). Species-specific alignments where then used to estimate p-distances between COI nucleotide sequences using the software MEGA 5.1 [45]. This was done for within-taxon divergence estimates based on i) pairwise p-distances between COI nucleotide sequences of each specimen and ii) average within group standard error obtained using 500-bootstrap replicates. Similarly, between-taxa average p-distances where calculated using the same software package and using 500 bootstrap replicates to obtain standard error. The same alignments were used to infer haplotype networks using popart (http://popart.otago.ac.nz) and the statistical parsimony network implementation that uses TCS [46, 47]. Popart depicts not only phylogenetic relationships but also haplotype frequencies origins, facilitating tests for haplotype sharing between different localities.

### Population genetics

Population specific parameters reflecting diversity and demography at the mtCOI gene were estimated using the software DnaSPv5 [48] for populations with at least 4 specimens. Population diversity is described mainly by haplotype diversity (Hd) and nucleotide diversity (π) [49]. Haplotype diversity describes the chance to observe two different haplotypes when randomly sampling two specimens from a population. Thus, the parameter ranges from 0 (one haplotype is fixed) to 1 (every specimen has a different haplotype). Similarly, nucleotide diversity describes the chances of observing two different nucleotides at a given COI sequence position when randomly sampling two specimens. This indicates the extent to which haplotypes within a population differ in their nucleotide composition. We also present the ratio between nucleotide diversity at non-synonymous (πa) and synonymous (πs) sites.

Demographic history of populations was analyzed using the software DnaSPv5 [48] to estimate the parameters TajimaD, FuFs, R2 and the ragedness index. With these estimates, we could test the hypothesis of expansive population growth. The parallel and comparative analyses of all four parameters is recommended, as shown by theoretical investigations indicating different sensitivities of each parameter, partly depending on population size [50]. Generally, Tajima’s D [51], Fu’s Fs [52], and Rs [50] indicate whether populations show an excess of singleton mutations, i.e., a pattern expected at neutrally evolving genetic markers if a populations is not in mutation-drift equilibrium as a result of expansive population growth. The Ragedness index [53] reflects the mismatch distribution of pairwise nucleotide differences between haplotypes and is expected to be unimodal under an expansion scenario. Divergence between populations was estimated based on an Analyses of Molecular Variance according to the approach by Weir and Cockerham (1984) [54] and implemented in the software Arlequin [55, 56].

#### Coalescent simulations

Statistical performance analyses can give insights into how many gene fragments may be used in order to get robust estimates into patterns of gene flow. They were conducted by re-estimating demographic parameters using simulated population genetic datasets with controlled parameter values (pseudo-observed datasets). Simulations were completed with *fastsimcoal2* [57] under two different isolation with migration models characterized by symmetrical and asymmetrical migration rates, respectively. A graphical representation of these models can be found in S1 Fig. Pseudo-observed datasets were simulated for 20 diploid individuals per population and with four different lengths of neutral genomic fragments per dataset: 10, 100, 1000, and 10000 (S2 Table). We assume that dirivultid copepods, as most copepods, are composed of diploid cells [58]. One fragment corresponded to a 600bp region with mutation and recombination rates of 1x10^-8^ events/bp/generation. For each model and for each number of simulated fragments we simulated 20 pseudo-observed datasets that were used as input to the maximum-likelihood parameter estimation procedure implemented in *fastsimcoal2* [9]. In both models the diploid population sizes were set to 10000 individuals and kept constant over time, based on the observed mean abundance of 8 individuals per 64 cm^2^ of *Stygiopontius hispidulus*, *Aphotopontius mammillatus* and *A*. *limatulus* (see discussion on diversity below) and assuming a vent field size of 8 m^2^. Time of divergence (T_DIV_) was set to 1000 and 100000 generations. T_DIV_ of 1000 is an estimate based on presumably high generation time of copepods and recent bottleneck events due to major volcanic eruptions. For T_DIV_ = 1000 we assume ~33 copepod generations per year in an environment with temperatures of 20°C [59], and major volcanic eruption ~33 years ago, a realistic current day scenario for fast spreading centers [27]. T_DIV_ = 100 000 is an estimate based on lower generation time (assuming 10 copepod generations per year in an environment of 10°C; [59]) and major disturbance event 10 000 years ago. The migration rate was set to 0.0001 and corresponds to the proportion of individuals within a population that belonged to another population in the previous generation. Here the product Nm equals 1, indicating one migrating individual per generation.

## Results

### Species identification, molecular taxonomy and intraspecific DNA sequence variation

**Fig 3:**
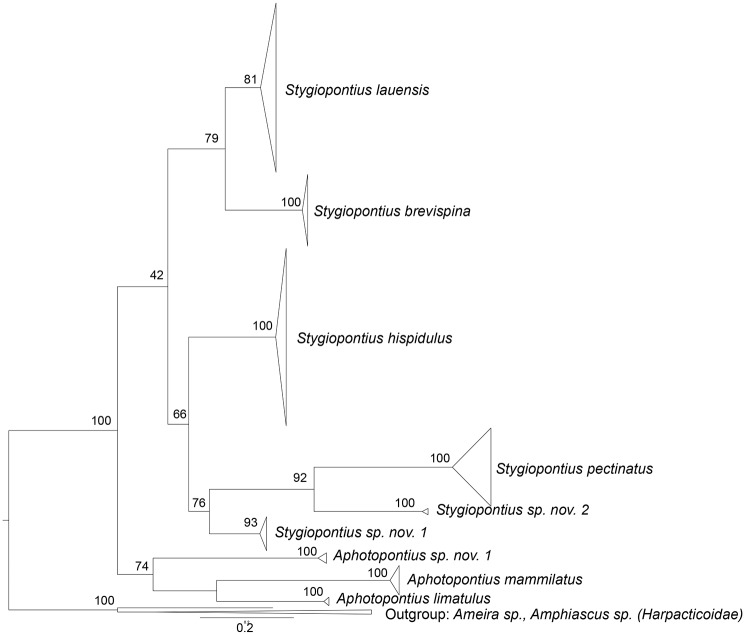
Maximum-likelihood phylogeny tree, calculated with RAxML tree and 1000 bootstraps, for the nine dirivultid copepod species on the basis of mtCOI data.

**Fig 4:**
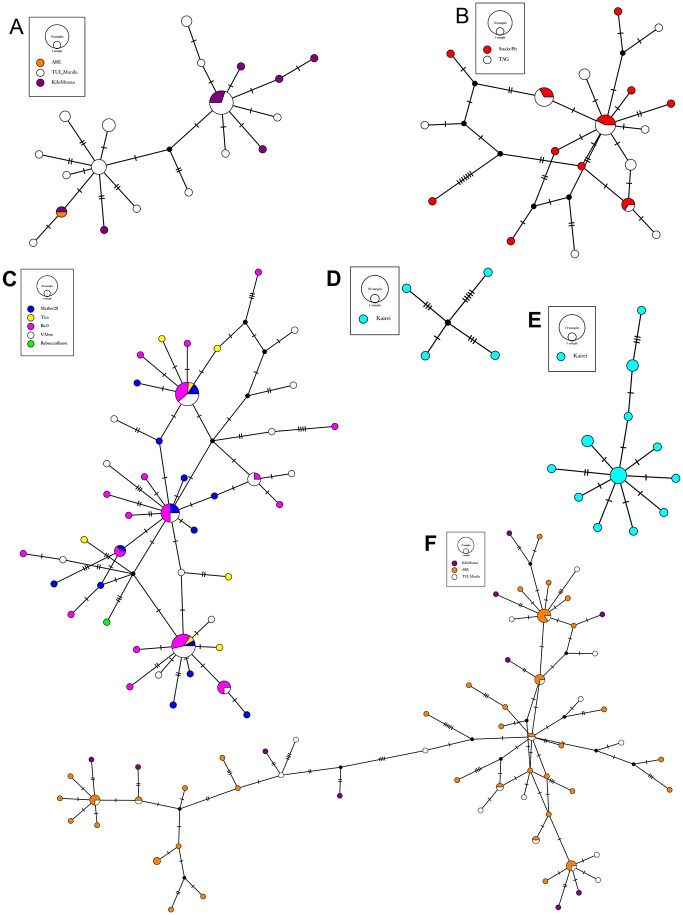
MtCOI haplotype networks of *Stygiopontius* species based on statistical parsimony. A. *S*. *brevispina* (ELSC), B. *S*. *pectinatus* (MAR), C. *S*. *hispidulus* (EPR, and one ind. from GC), D. *S*. sp. nov. 2 (CIR), E. S. sp. nov. 1 (CIR), F. *S*. *lauensis* (ELSC). Species occurred in different regions: Eastern Lau Spreading Center (ELSC), Mid Atlantic Ridge (MAR), East Pacific Rise (EPR), Gulf of California (GC) and Central Indian Ridge (CIR). Distinct haplotypes are depicted as circles (indicating haplotypes found at a single locality) and pie charts (haplotype found at different localities) with a diameter proportional to their frequency among all haplotypes found in each species. A color code indicates the sample site and the frequency distribution (pie charts) of haplotypes for each locality. Circles and pie charts are connected by lines whereas black dots and slashes indicate missing haplotypes (providing a relative estimate of genetic divergence between haploytypes).

**Fig 5:**
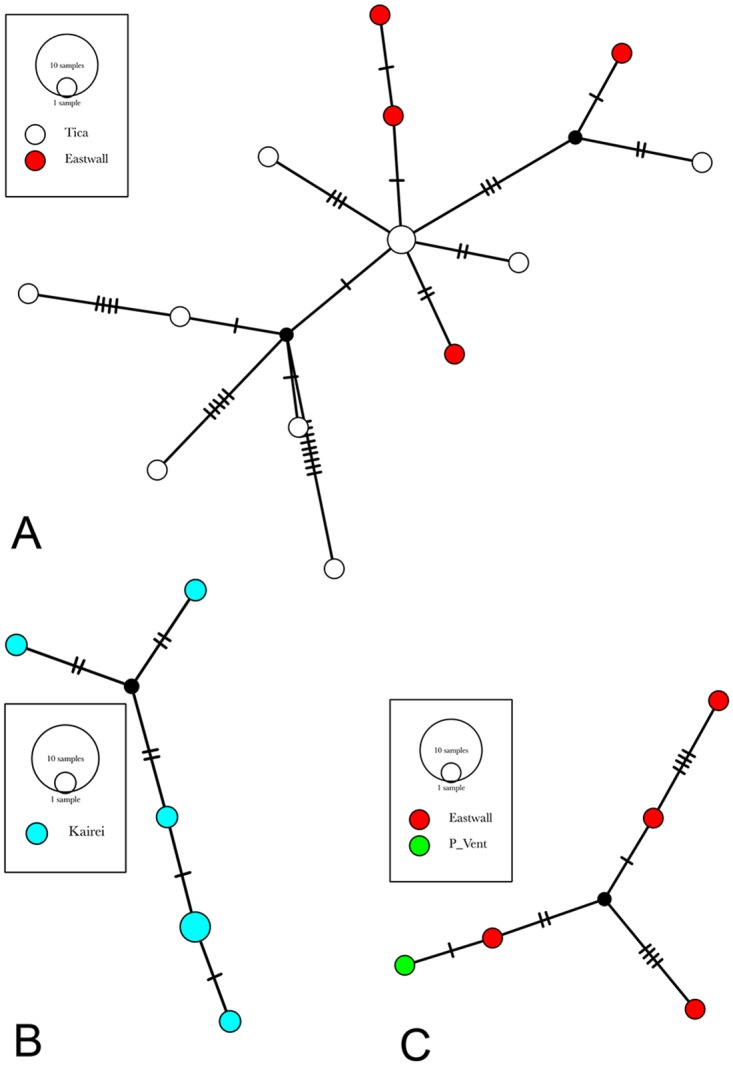
COI haplotype networks of *Aphotopontius* species based on statistical parsimony. A. *A*. *mammillatus* (EPR), B. *A*. sp. nov. 1 (CIR), C. *A*. *limatulus* (EPR). Species occurred in different regions: East Pacific Rise (EPR), and Central Indian Ridge (CIR). Distinct haplotypes are depicted as circles (indicating haplotypes found at a single locality) and pie charts (haplotype found at different localities) with a diameter proportional to their frequency among all haplotypes found in each species. A color code indicates the sample site and the frequency distribution (pie charts) of haplotypes for each locality. Circles and pie charts are connected by lines whereas black dots and slashes indicate missing haplotypes (providing a relative estimate of genetic divergence between haploytpes).

Morphological identification and maximum-likelihood phylogenetic analyses of mitochondrial COI sequences (~650 bp) revealed the same entities and there was no evidence for cryptic species (Fig 3). Uncorrected p-distances between species ranged from 15 to 28% within the genus *Stygiopontius*, and from 23 to 29% within the genus *Aphotopontius*. Intraspecific p-distance estimates were low for *Stygiopontius spec*. (0.5–1,4%) and *Aphotopontius* spec. (0.8–1.2%) (Table 2). The maximum-likelyhood phylogeny groups all *Stygiopontius* species. *S*. *lauensis* and *S*. *brevispina* from ELSC (bootstrap value XY79) from a cluster. *S*. sp. 1 nov., *S*. sp.2 nov. from CIR from a cluster with S. pectinatus from MAR (bootstrap value 76). Haplotype networks of *Stygiopontius* spp. (Fig 4) and *Aphotopontius* spp. (Fig 5) indicate that intraspecific nucleotide differences between haplotypes are small and haplotypes are related by only few mutational steps (Figs 4 and 5). There is no correlation between topology and distribution of haplotypes among localities, and haplotype sharing among localities.

**Table 2:** Genetic p-distances referring to taxa and their respective clusters as derived in Maximum-Likelihood phylogenetic inference.

Taxon	intraspecific p-distances				interspecific p-distances	
	min	max	mean	SE	min	max
*Stygiopontius sp*.*nov*. *1*	0	0.0136	0.0048	0.0012	0.1784	0.2727
*Stygiopontius sp*.*nov*. *2*	0.0066	0.0148	0.0109	0.0029	0.2049	0.2956
*Stygiopontius pectinatus*	0	0.06	0.0103	0.0015	0.2049	0.3101
*Stygiopontius hispidulus*	0	0.018	0.0062	0.0015	0.1784	0.2877
*Stygiopontius lauensis*	0	0.0319	0.0138	0.0024	0.1461	0.2755
*Stygiopontius brevispina*	0	0.0102	0.0051	0.0013	0.1461	0.2974
*Aphotopontius sp*.*nov*. *1*	0.0016	0.0193	0.0108	0.0027	0.2179	0.2974
*Aphotopontius limatulus*	0.0016	0.0128	0.0083	0.0025	0.2311	0.3101
*Aphotopontius mammillatus*	0	0.0313	0.0117	0.0018	0.2311	0.3101

Within group (intraspecific) parameter range refers to pairwise p-distance estimates between individual mtCOI-nucleotide sequences. Within group means are given with Standard Error (SE) that was calculated based on 500 bootstrap replicates. Between group (interspecific) means are listed as minimum and maximum mean values and the complete set of pairwise comparisons is given in S3 Table.

### Diversity and Demography

Diversity and demography parameters were estimated for populations with at least 4 specimens (see Table 1). Haplotype diversity was high among all *Stygiopontius* spp., ranging from 0.893 to 1.00 and nucleotid diversity ranged from <0.001 to 0.01 (Table 3) Values for both diversity estimates were in the same range for the three *Aphotopontius* spp. (Hd ranging between 0.8 and 1.0; π: 0.008–0.01) (Table 3). The ratio between nucleotide diversity at non-synonymous and synonymous sites (πa/πs) was much less than one for all taxa.

**Table 3:** Species specific diversity (within populations) and divergence (between populations) parameters estimates based on COI sequences.

Species	Region	Site	N_Seq_	Sites_used/_Sites_alignment_	N_Hap_	Hd	S	π	π_a_/π_s_	AMOVA
*Stygiopontius sp*.*1*	CIR	Kairei	17	477/625	12	0.941	14	0.00465	0.018	-
*Stygiopontius sp*.*2*	CIR	Kairei	4	540/612	4	1	12	0.0112	0.061	-
*Stygiopontius pectinatus*	MAR	Snake Pit	15	435/660	11	0.952	20	0.00893	0.064	-
		TAG	18	504/666	12	0.935	21	0.00695	0.033	-
		Turtles Pit	2	-	2	1	-	-	-	-
		overall	33	435/666	18	0.922	27	0.00723	0.04	0.07 (P = 0.051)
*Stygiopontius hispidulus*	EPR	Marker 28	16	657/657	14	0.983	19	0.0061	0	-
		Tica	7	657/657	7	1	11	0.00656	0.023	-
		Bio9	32	657/657	19	0.946	30	0.00655	0.02	-
		V-Vent	27	655/657	15	0.917	22	0.00529	0	-
		Rebeccas Roost	1	-	-	-	-	-	-	-
		overall	83	655/657	44	0.942	52	0.00556	0.011	-0.00141 (P = 0.472)
*Stygiopontius lauensis*	ELSC	ABE	50	657/657	40	0.985	61	0.01442	0.003	-
		TU'i Malila	23	639/657	23	1	38	0.0125	0	-
		Kilo Moana	7	618/657	7	1	26	0.01718	0.008	-
		overall	80	600/657	60	0.986	73	0.01483	0.003	0.02026 (P = 0.146)
*Stygiopontius brevispina*	ELSC	ABE	1	-	-	-	-	-	-	-
		TU'i Malila	26	639/657	15	0.945	20	0.00514	0	-
		Kilo Moana	8	588/657	6	0.893	8	0.00372	0	-
		overall	35	571/657	19	0.906	23	0.00483	0	0.11027 (P = 0.02)
*Aphotopontius sp*. *1*	ELSC	Kairei	6	459/648	5	0.933	8	0.00731	0	-
*Aphotopontius limatulus*	EPR	Kairei	6	459/648	5	0.933	8	0.00731	0	-
		Eastwall	4	624/669	4	1	10	0.0086	0	-
		P-Vent	1	-	1	-	-	-	-	-
		overall	5	624/669	5	1	11	0.00839	0	-
Aphotopontius mammillatus	EPR	Tica	10	528/675	9	0.978	30	0.01281	0.269	-
		Eastwall	4	660/675	4	1	10	0.00788	0	-
		overall	14	528/675	13	0.989	34	0.01161	0.18	-0.06988 (P = 0.8522)

Diversity parameters are given for each locality: N_Seq_ = Numbers of COI sequences, Sites_used_ = Number of sites that are available for each specimen within the alignment, Sites_alignment_ = Total length of the alignment, N_Hap_ = Number of haplotypes, Hd = Haplotype diversity, S = Number of segregating sites, π = Jukes-Cantor corrected estimate of nucleotide diversity, π_a_/π_s_ = ratio of Jukes-Cantor corrected estimates for nucleotide diversity at non-synonymous nucleotide positions (π_a_) and nucleotide diversity at synonymous nucleotide positions (π_s_). Divergence estimates are based on an analysis of molecular variance (AMOVA) and where performed for all species found at different sites (F_st_ values with a P-value derived from bootstrapping). Calculation where only performed for sites with at least 4 nucleotide sequences.

Demography parameter estimates indicated that all populations are undergoing growth (Table 4). Tajima’s D was always negative, indicating an excess of rare alleles (i.e. singleton mutations), and ranged from -0.8 to -2.1 for *Stygiopontius* spp. from all biogeographic regions. Some of the more extreme values were seen for *S*. *pectinatus* from MAR (-1.9; p < 0.05), for *S*. *brevispina* from ELSC (-1.8; p < 0.05), and for *S*. *hispidulus* from EPR (-2.1; p < 0.05). Similarly, Fu’s F values were significantly negative, and R2 and raggedness index (rg) were low for *Stygiopontius* species, indicating sudden expansion. Similar values (negative Tajima D and Fu’s F; low R2 and rg) were observed for *Aphotopontius* species (Table 4). This excess of rare alleles in all taxa is also reflected in haplotypes networks (Figs 4 and 5), which indicate a majority of singleton haplotypes.

**Table 4:** Parameters describing demography of species specific populations found at different sites.

Species	Region	Site	N_Seq_	N_Hap_	Tajima D	Fu Fs*	R2*	rg*
*Stygiopontius sp*.*1*	CIR	Kairei	17	12	-1.786 (n.s.)	-8.411 (P<0.0001)	0.068 (P<0.0001)	0.1094 (P = 0.4072)
*Stygiopontius sp*.*2*	CIR	Kairei	4	4	-0,851 (n.s.)	-0.288 (P = 0.2566)	0.1179 (P = 0.0275)	0.6667 (P = 0.7929)
*Stygiopontius pectinatus*	MAR	Snake Pit	15	11	-1.6621 (n.s.)	-4.481 (P = 0.014)	0.0946 (P = 0.033)	0.0405 (P = 0.1300)
		TAG	18	10	-1.6851 (n.s.)	-5.117 (P<0.0001)	0.0878 (P = 0.0320)	0.0356 (P = 0.092)
		Turtles Pit	2	-	-	-	-	-
		overall	33	18	-1.9359 (P<0.05)	-10.113 (P<0.0001)	0.057 (P = 0.010)	0.0335 (P = 0.1170)
*Stygiopontius hispidulus*	EPR	Marker 28	16	14	-1.2132 (n.s.)	-9.291 (P<0.0001)	0.0799 (P = 0.003)	0.0338 (P = 0.089)
		Tica	7	7	-0.2458 (n.s.)	-3.398 (P = 0.011)	0.1423 (P = 0.088)	0.0635 (P = 0.0983)
		Bio9	32	19	-1.6559 (n.s.)	-8.867 (P = 0.001)	0.0678 (P = 0.048)	0.0285 (P = 0.114)
		V-Vent	27	15	-1.4221 (n.s.)	-6.431 (P = 0.003)	0.0649 (P = 0.011)	0.0379 (P = 0.1270)
	GC	Rebeccas Roost	1	1	-	-	-	-
		overall	83	44	-2.1433 (P<0.05)	-44.459 (P<0.0001)	0.0300 (P = 0.001)	0.0174 (P = 0.030)
*Stygiopontius lauensis*	ELSC	ABE	50	40	-1.1712 (n.s.)	-26.303 (P<0.0001)	0.0677 (P = 0.080)	0.0087 (P = 0.023)
		TU'i Malila	23	23	-0.8991 (n.s.)	-18.459 (P<0.0001)	0.0852 (P = 0.065)	0.0101 (P = 0.011)
		Kilo Moana	7	7	-0.2807 (n.s.)	-1.479 (P = 0.124)	0.1271 (P = 0.052)	0.0726 (P = 0.2020)
		overall	80	60	-1.3987 (n.s.)	-34.502 (P<0.0001)	0.0546 (P = 0.057)	0.0050 (P = 0.005)
*Stygiopontius brevispina*	ELSC	ABE	1	-	-	-	-	-
		TU'i Malila	26	15	-1.3499 (n.s.)	-7.124 (P = 0.002)	0.071 (P = 0.019)	0.0395 (P = 0.135)
		Kilo Moana	8	6	-1.4213 (n.s.)	-2.401 (P = 0.016)	0.1608 (P = 0.133)	0.111 (P = 0.2059)
		overall	35		-1.8232 (P<0.05)	-12.601 (P<0.0001)	0.0508 (P = 0.001)	0.0257 (P = 0.043)
*Aphotopontius sp*. *1*	ELSC	Kairei	6	5	-0.2866 (n.s.)	-1.082 (P = 0.161)	0.1531 (P = 0.677)	0.1644 (P = 0.3212)
*Aphotopontius limatulus*	EPR	Eastwall	4	4	-0.2223 (n.s.)	-0.439 (P = 0.2295)	0.1394 (P = 0.0428)	0.1667 (P = 0.1294)
		P-Vent	1	1	-	-	-	-
		overall	5	5	-0.1091 (n.s.)	-1.283 (P = 0.1168)	0.1640 (P = 0.1113)	0.1400 (P = 0.1901)
Aphotopontius mammillatus	EPR	Tica	10	9	-1.8734 (P<0.05)	-2.683 (P = 0.064)	0.0936 (P = 0.014)	0.0627 (P = 2760)
		Eastwall	4	4	-0.5281 (n.s.)	-0.480 (P = 0.222)	0.1816 (P = 0.1265)	0.333 (P = 0.483)
		overall	14	13	-2.0096 (P<0.05)	-6.624 (P<0.0001)	0.0757 (P = 0.003)	0.0315 (P = 0.1090)

Estimates where performed for site specific population with a minimum size of 4 specimens and estimates are given for four different estimators with p-values given in parentheses. rg = Raggedness index, *Test for significance based on a coalescence approach using 1000 replicates, assuming no recombination and given the estimate for Theta under the Population growth-decline model as implemented in DNASP; P indicates the probability to observe a value larger or equal to the observed value.

### Divergence

AMOVA-based estimates of divergence between populations were made for species that where found at more than one locality (Table 3). We estimate *F*_*ST*_ as 0.07 (p = 0.05) for *S*. *pectinatus* from MAR sites (sites > 300 km apart). At ELSC sites, *F*_*ST*_ was 0.02 (p = 0.15) for *S*. *lauensis* and 0.11 (p = 0.02) for *S*. *brevispina*; this includes samples from three vent sites separated by 80 and 140 km. In contrast, EPR vent sites were separated by just a few hundred meters and at most by 5 km, so estimates for *S*. *hispidulus* were only -0.001 (p = 0.47) (Table 2). Low divergence between sites is also reflected in the haplotype networks (Figs 4 and 5) as there is no association between network topology and sampling locality. Apart from *Stygiopontius* species, divergence estimates were also possible for *Aphotopontius mammillatus* at EPR. Low population divergence (*F*_*ST*_t = -0.07, p = 0.9) also reflected by the absence of a relationship between network topology and sampling locality.

### Impact of the number of sequenced fragments on the statistical performance of demographic estimations

**Fig 6:**
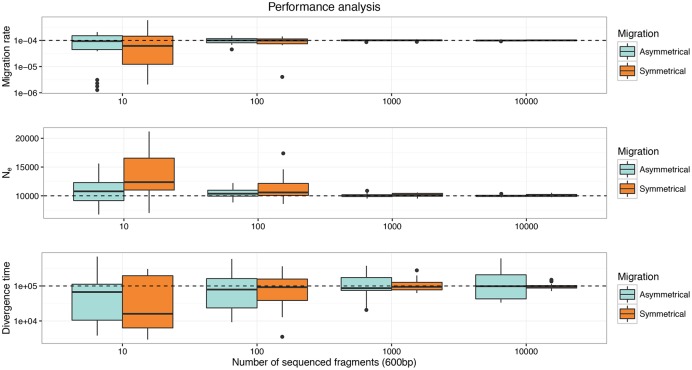
Performance analyses of *fastsimcoal2* assuming a divergence time (T_DIV_) of 100000 generations for different numbers of sequenced fragments in isolation with migration models. In every panel the dashed horizontal line represents the true value of the parameter and the boxplots represent 20 re-estimations of these parameters under the correct model.

**Fig 7:**
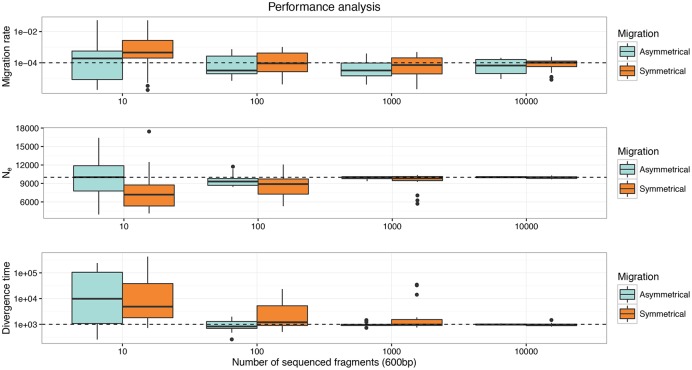
Performance analyses of *fastsimcoal2* assuming a divergence time (T_DIV_) of 1000 generations for different numbers of sequenced fragments in isolation with migration models. In every panel the dashed horizontal line represents the true value of the parameter and the boxplots represent 20 re-estimations of these parameters under the correct model.

Figs 6 and 7 summarize the results of the performance analysis of demographic estimations with varying numbers of sequenced fragments and assuming population divergence times (T_DIV_) of 100000 and 1000 generations. Results for T_DIV_ = 100000 show that accurate estimates of the migration rate and the population size can be achieved when the number of simulated fragments is at least 100 both for the symmetrical and asymmetrical migration models. The reason for the low performance in re-estimating the age of the split between the two populations in the asymmetrical migration rate model can be explained by the fact that the time to the most recent common ancestor in the whole sample is probably consistently younger than the age of the population split (100000 generations). Analyses with a divergence time of 1000 generations provided similar results: using at least 100 fragments gives good estimates on migration rate although precision in the re-estimation of the migration rate decreased compared to the case where T_DIV_ = 100000 (Figs 6 and 7). Overall, our results show that a dataset composed of 100 fragments sequenced in two population samples of 20 diploid individuals allows gaining insight into patterns of gene flow in these deep sea populations.

## Discussion

### Species identification and molecular taxonomy

Our DNA sequences of dirivultid copepods confirmed the morphologically identified species and showed that mtCOI is a powerful tool to identify dirivultid species. The protocol developed earlier for species from EPR and ELSC [39] worked very well here for species from MAR and CIR. Net distances between newly analyzed dirivultid species were similar to what was observed earlier, and previously morphologically undescribed dirivultid species had similar distances as described ones [39]. Thus, morphological and molecular taxonomy matches very well within Dirivultidae, and there was no indication of cryptic species, as also indicated by the haplotype networks.

### Diversity

High haplotype diversity of dirivultid copepods corresponds to high site occupancy, similar to what was observed for macrofauna species from EPR [14]. Copepod specimens used for mtCOI analyses from 9°N EPR were collected from 2006 to 2009 and come partly from the same collections (often the same sites) as a community recovery of fauna after a major volcanic eruption in this area in 2006 [22]. *Stygiopontius hispidulus*, *Aphotopontius mammillatus*, and *A*. *limatulus* were present in the majority of these community samples, and were highly abundant locally (e.g. 239 *S*. *hispidulus* ind. per 64 cm^2^ at site Bio 9 in 2006; 124 *A*. *limatulus* ind. per 64 cm^-2^ at site Eastwall in 2009; 29 *A*. *mammillatus* ind. per 64 cm^-2^ at site Tica in 2009; for mean values see S4 Table). The mean abundance of these three species was 8 ind. per 64 cm^-2^ (corresponding to the size of artificial settlement device used by Gollner et al. [22]), and thus about 1250 individuals per species per square meter can be expected in this region. Thus, a vent field with a size of ~50 m^2^ could harbor more than 60 000 individuals per dirivultid species in this EPR region. Dirivultid species from ELSC were also abundant and inhabited all three sites studied (ongoing study Gollner et al.). *S*. *pectinatus* from MAR is known to occur in high abundances in close association with the shrimp *Rimicaris exoculata* (pers. obs. Florence Pradillion IFREMER). To conclude, Dirivultids maintain numerous colonies with locally abundant populations, characterized genetically by high haplotype diversity.

Large dirivultid population sizes might be a result of high resource availability at hydrothermal vents. Most dirivultid copepods are deposit feeders and/or graze on the abundant bacterial mats associated with hydrothermal vents [4, 60, 61]. Limen et al. [62] observed that Dirivultids even show food partitioning within the same trophic level [63]. In addition to this nutritional adaptation, dirivultid copepods exhibit high hemoglobin concentrations which may help them to up take oxygen and to thrive in the low oxygen vent environments [64, 65]. Also, they are typically faster and larger than other vent meiofauna [1, 32]. Thus, Dirivultids are highly competitive in the vent environment, which could allow them to take particular advantage of the high food concentrations in the extreme vent environment and hence grow to large population sizes, finally leading to high haplotype diversity.

The copepod species studied here live in close association with highly abundant megafauna that show similarly high haplotype diversity. For example, the copepod *S*. *pectinatus* is found associated with the shrimp *Rimicaris exoculata*. It lives around and inside the gills of the shrimp and likely grazes on the bacteria farmed there [66, 67]. Haplotype diversity of *R*. *exoculata* ranged from 0.69 to 0.82 for sample populations with more than 11 individuals, and was similarly high as in *S*. *pectinatus*, with values of with 0.94 to 1 [15]. The copepod *S*. *hispidulus* (hd 0.92–1) is typically found in very high abundance and in association with the pompei worm *Alvinella pompejana* [33]. Haplotype diversity of the Pompeii worm was also high at 0.91 [68]. *S*. *lauensis* (Hd 0.98–1) was very abundant amongst the snail *Ifremeria nautilei* (pers. obs. Sabine Gollner) with Hd ranging from 0.61 to 1 [69]. These findings suggest that high site occupancy can result in high haplotype diversity regardless of traits such as size (meiofauna versus megafauna), mobility (e.g. highly mobile copepods in versus limited vagility of *I*. *nautilei*), larval nutrition (lecitotrophic Dirivultid larvae versus planctotrophic *R*. *exoculata* larvae), or number of offspring per individual (4 per dirivultid copepod, many for megafauna) [14].

### Genetic Connectivity

We observed similar genetic connectivity within the same genus regardless of distinct field frequency on EPR, ELSC, and MAR. This conclusion was based on divergence estimates derived from our AMOVA analyses, i.e., F_ST_ values are generally low and non-significant indicating weak or no divergence between populations at different vent sites. In addition, haplotype networks showed no correlation between topology and distribution of haplotypes, and haplotype sharing among localities, pointing to similar phylogeography of species and unrestricted gene-flux during colonization processes. A priori, we had expected that genetic connectivity within dirivultids having similar life history traits is higher at fast-spreading centers with many vent fields (one every 10–20 km) than at slow-spreading centers with few vent fields (one every 100 km) [26]. High vent field frequency supports a larger effective size of a metapopulation and hence the number of colonies that supply migrants [14]. We speculate that connectivity is also influenced by frequency of natural disturbance events: on fast spreading centers, frequent volcanic eruptions might frequently kill entire local populations and thus diminish connectivity, whilst rare volcanic eruptions on slow spreading centers might have very little effect. Thus, the two geological forces of vent field frequency and disturbance rate could act conversely on connectivity of dirivultid populations in slow- and fast-spreading centers, resulting in similar connectivity.

Ocean current regimes also play a crucial role in vent fauna connectivity and dispersal, since larvae typically drift passively. Dirivultids have lectitotrophic nauplii [36, 37] and feeding copepodites that both have been observed in the pelagial above vents [22, 38]. Mullineaux et al. 2005 showed that larval abundances of vent gastropods, polychaetes, a bivalve and a crab were significantly higher on-vent than off-vent at the 9° NEPR, suggesting that larvae may be retained within the valley [70]. Fracture zones may channel deep water currents in such a way that they act as barriers to passive larval drift [71]. For this study, we have too little information to estimate role of current regimes on dirivultid dispersal in the distinct geographical settings. However, hydrodynamic modeling and Lagrangian particle tracking could help to estimate dispersal distances and directions in the future [21].

Connectivity between active vent sites might be also accomplished through the use of intermediate habitats as stepping stones. Vent copepods have been observed, although not frequently, up to 1 kilometer away from the nearest vent [1, 38]. Nematode species typically associated with active vents have also been observed at inactive vent sites lacking vent fluid supply for more than four years [30]. Whether such habitats can support large enough dirivultid populations to substantially contribute to connectivity between vent sites is currently unclear.

Biological traits may also influence species connectivity. High gene flow in the shrimp *Rimicaris exoculata*, even along the slow spreading MAR, was likely related to several phenotypic and life history traits: its very large lecitotrophic eggs, planktotrophic larvae that feed in photic zones, delayed metamorphosis, and active, directed migration [14, 15, 20]. The pompei worm *Alvinella pompejana* from fast-spreading EPR likely experiences high gene flow rates due to its lecitotrophic larvae (which arrest development in cold bottom waters), prolonged larval duration and increasing dispersal potential [18, 19, 68, 72]. Studies on larval duration and either potential or realized dispersal of dirivultid copepods are currently lacking, but such studies may help estimate the influence of biological traits on connectivity.

### Demography

The negative Tajima's D values observed in our dataset are consistent with population size expansion, or could reflect the effects of background selection acting on a non-recombining genome [73]. Population expansion among all Dirivultids could be explained by the food-rich vent environment (see discussion on diversity and population size above), which allows copepods to produce many offspring. Expanding populations could also foster survival in the temporally unstable vent environment. At 9°N EPR local eruptions occur approximately every 15 years [29], and kill almost all fauna in the region [22, 23]. In addition, vent sites frequently vein, often within only a few months or years [74]. This instability could cause frequent local bottlenecks, resulting in decreased genetic variability. However, variability could quickly rebound, given the rapidly expanding copepod populations. Indeed, as many as 30 individuals per 1000 liters was detected at three meters above vent bottom after the volcanic eruption in 2006 [22]. This is even greater than values reported for gastropod and polychaete larvae in the same area at four meters above bottom (ranges from 2–11 ind. per 1000 l) [75]. In addition, meiofauna typically have more generations per year (averaging three) [76]; futher, Huntley et al. (1991) found that generation time is a factor of temperature in copepods, recording average generation times of 125 days at 2°C, of 25 days at 15°C, and of only 10 days at 25°C [77]. Temperature was generally hot but also quite variable in the habitats were we sampled, ranging from 14 to 110°C among Pompeii worm collections, for example [33, 78]. Thus, we expect that dirivultid copepods likely show short generation times of several days or weeks. We speculate that dirivultid populations actively invest in expansion and release their offspring into the pelagial to colonize new vents, thus preventing sudden extinction via volcanic eruptions or veining of vent sites.

## Conclusion

Our comparative study suggests that populations of dirivultid copepods show a pattern of generally high haplotype diversity, high connectivity and population expansion, regardless of vent region. On the EPR, high species abundance values in the benthos and in the pelagial support our genetic data. Benthic copepod abundance data from CIR and ELSC are currently being analyzed by TK and SG, with first results (not yet published) implying that these also support our genetic data. Dirivultids maintain numerous colonies in close proximity, with locally abundant populations, allowing them to quickly colonize nascent vents. This could make these copepods rather robust against threats of mineral mining, such as destruction of active vent sites. However, it should be acknowledged that Dirivultids have different, important characteristics compared to other vent copepods and meiofauna taxa [1]. For example, within the meiofauna size class, only dirivultid copepods were observed frequently in the pelagial, while all other meiofauna taxa were rare [22]. We expect that other vent meiofauna taxa will show less connectivity compared to dirivultid copepods.

Our mtDNA results also raised questions that need to be addressed in the future in order to better understand the underlying processes of connectivity and population expansion. Genomic approaches analyzing 100 fragments, as well as current regime studies and studies on life history traits such as larval duration can help to further unravel mechanisms leading to high genetic connectivity in Dirivultids in areas of different vent field frequencies. These more detailed results will be necessary to the design of deep-sea preserves in areas that are targeted for future mineral mining operations.

## Supporting Information

S1 Fig The two demographic models used or the performance analyses. Both models are characterized by a population size of 10000 diploid individuals, a time of divergence of 100000 and 1000 generations respectively, and a migration rate of 0.0001. The model on the left allows migration to occur at rate 0.0001 in both directions whereas the model on the right only allows migration to occur in a single direction.
(TIF)

S1 Table Gen Bank accession number of Dirivultid specimens (mtCOI-nucleotide sequences) used for comparative analyses of genetic structure in hydrothermal vent populations. All these samples passed i) quality check of Sanger Sequence-Electropherograms, ii) Blast search if reads for artifacts (e.g. human COI), iii) can be unambiguously translated into a COI-amino acid sequence, and iv) clustered in reciprocally monophyletic clades according to their *a priori* classification based on their morphological characters. Sequences that did not fulfill these four criteria were completely excluded. Species name, individual identification number, locality, source, phylogenetic assignment, and usage in population genetics are given. Genera: *A*.*–Aphotopontius; S*.*–Stygiopontius*.
(PDF)

S2 Table Simulated datasets that were used as input to the maximum-likelihood parameter estimation procedure implemented in *fastsimcoal2*. Identification number of simulation (id), divergence time in generations (tau), mutation and recombination rates of 1x10^-8^ events/per bp/ per generation (m), number of individuals per population (Ne), number of migrants per generation (migr./gen.), length of loci in base pairs (bp), mjgration type, number of pseudo observed data (pseu.obs.), and number of replicates per pseudo observed sample (repl.pseu.obs.).
(PDF)

S3 Table Average p-distance between taxa (below diagonal) and respective standard error calculations based on 500 bootstrap replicates (above diagonal). (PDF)

S4 Table Mean copepod abundance per 64 cm^2^ in artificial settlement devices used to study recovery of fauna on the 9°N East Pacific Rise after the 2006 eruption. Site, year of collection, number of samples (n), and species are given. Abundance data from sites Sketchy, P-Vent and Tica were extracted from raw data from Gollner et al. 2015. Data from sites Bio9 and Eastwall are here published for the first time. Information on sampling strategies and methods in Gollner et al. 2013 and Gollner et al. 2015.
(PDF)
