# Supplementary figures and images for: Mitochondrial DNA Analyses Indicate High Diversity, Expansive Population Growth and High Genetic Connectivity of Vent Copepods (Dirivultidae) across Different Oceans
Source: PLoS One. 2016 Oct 12;11(10):e0163776. doi: 10.1371/journal.pone.0163776 (PMC5061364; doi:10.1371/journal.pone.0163776)

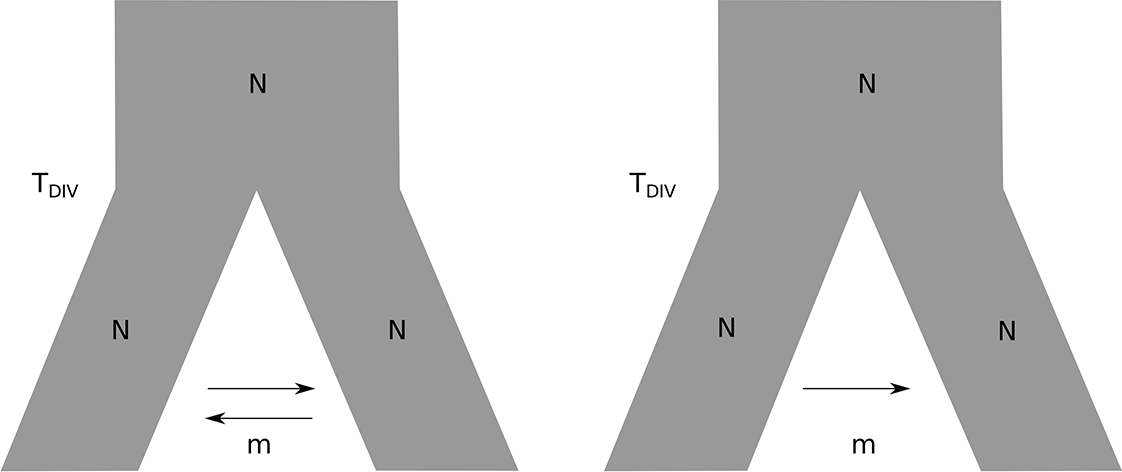

Supplement: S1 Fig — Both models are characterized by a population size of 10000 diploid individuals, a time of divergence of 100000 and 1000 generations respectively, and a migration rate of 0.0001. The model on the left allows migration to occur at rate 0.0001 in both directions whereas the model on the right only allows migration to occur in a single direction. (TIF) [file pone.0163776.s001.tif]
