# Supplementary material for: Mitochondrial DNA Analyses Indicate High Diversity, Expansive Population Growth and High Genetic Connectivity of Vent Copepods (Dirivultidae) across Different Oceans
Source: PLoS One. 2016 Oct 12;11(10):e0163776. doi: 10.1371/journal.pone.0163776 (PMC5061364; doi:10.1371/journal.pone.0163776)
Supplement: S1 Table — All these samples passed i) quality check of Sanger Sequence-Electropherograms, ii) Blast search if reads for artifacts (e.g. human COI), iii) can be unambiguously translated into a COI-amino acid sequence, and iv) clustered in reciprocally monophyletic clades according to their a priori classification based on their morphological characters. Sequences that did not fulfill these four criteria were completely excluded. Species name, individual identification number, locality, source, phylogenetic assignment, and usage in population genetics are given. Genera: A.–Aphotopontius; S.–Stygiopontius. (PDF) [file pone.0163776.s002.pdf]

S1 Table. **Gen Bank accession number of Dirivultid specimens (mtCOI-nucleotide sequences) used for comparative analyses of genetic structure in hydrothermal vent populations.** All these samples passed i) quality check of Sanger Sequence-Electropherograms, ii) Blast search if reads for artifacts (e.g. human COI), iii) can be unambiguously translated into a COI-amino acid sequence, and iv) clustered in reciprocally monophyletic clades according to their *a priori* classification based on their morphological characters. Sequences that did not fulfill these four criteria were completely excluded. Species name, individual identification number, locality, source, phylogenetic assignment, and usage in population genetics are given. Genera: *A. – Aphotopontius; S. – Stygiopontius.*

Species	ID	Region	Site	Source	Usage in pop. gen.	Gen Bank #
A. sp.nov. 1	I13_03	CIR	Karei	this study	yes	KX714826
A. sp.nov. 1	I13_13	CIR	Karei	this study	yes	KX714827
A. sp.nov. 1	I13_17	CIR	Karei	this study	yes	KX714828
A. sp.nov. 1	I13_19	CIR	Karei	this study	yes	KX714825
A. sp.nov. 1	I13_20	CIR	Karei	this study	yes	KX714829
A. sp.nov. 1	I13_21	CIR	Karei	this study	yes	KX714824
S. sp. nov. 1	I13_12	CIR	Karei	this study	yes	KX714856
S. sp. nov. 1	I13_27	CIR	Karei	this study	yes	KX714867
S. sp. nov. 1	I13_25	CIR	Karei	this study	yes	KX714857
S. sp. nov. 1	I13_28	CIR	Karei	this study	yes	KX714864
S. sp. nov. 1	I13_29	CIR	Karei	this study	yes	KX714868
S. sp. nov. 1	I13_30	CIR	Karei	this study	yes	KX714858
S. sp. nov. 1	I13_31	CIR	Karei	this study	yes	KX714865
S. sp. nov. 1	I13_37	CIR	Karei	this study	yes	KX714866
S. sp. nov. 1	I13_39	CIR	Karei	this study	yes	KX714869
S. sp. nov. 1	I13_40	CIR	Karei	this study	yes	KX714859
S. sp. nov. 1	I13_41	CIR	Karei	this study	yes	KX714863
S. sp. nov. 1	I13_42	CIR	Karei	this study	yes	KX714862
S. sp. nov. 1	I13_43	CIR	Karei	this study	yes	KX714854
S. sp. nov. 1	I13_44	CIR	Karei	this study	yes	KX714855
S. sp. nov. 1	I13_46	CIR	Karei	this study	yes	KX714870
S. sp. nov. 1	I13_48	CIR	Karei	this study	yes	KX714861

S. sp. nov. 1	I13_50	CIR	Karei	this study	yes	KX714860
S. sp.nov. 2	I13_26	CIR	Karei	this study	yes	KX714850
S. sp.nov. 2	I13_23	CIR	Karei	this study	yes	KX714852
S. sp.nov. 2	I13_32	CIR	Karei	this study	yes	KX714853
S. sp.nov. 2	I13_52	CIR	Karei	this study	yes	KX714851
S. pectinatus	PL5-SP-P56	MAR	Snake Pit	this study	yes	KX714897
S. pectinatus	PL5-SP-P57	MAR	Snake Pit	this study	yes	KX714873
S. pectinatus	PL5-SP-P58	MAR	Snake Pit	this study	yes	KX714898
S. pectinatus	PL5-SP-P59	MAR	Snake Pit	this study	yes	KX714899
S. pectinatus	PL5-SP-P60	MAR	Snake Pit	this study	yes	KX714896
S. pectinatus	PL5-SP-P61	MAR	Snake Pit	this study	yes	KX714872
S. pectinatus	PL5-SP-P62	MAR	Snake Pit	this study	yes	KX714900
S. pectinatus	PL5-SP-P64	MAR	Snake Pit	this study	yes	KX714895
S. pectinatus	PL5-SP-P65	MAR	Snake Pit	this study	yes	KX714876
S. pectinatus	PL5-SP-P66	MAR	Snake Pit	this study	yes	KX714901
S. pectinatus	PL5-SP-P67	MAR	Snake Pit	this study	yes	KX714894
S. pectinatus	PL5-SP-P69	MAR	Snake Pit	this study	yes	KX714893
S. pectinatus	PL5-SP-P70	MAR	Snake Pit	this study	no, too short	KX714877
S. pectinatus	PL5-SP-P71	MAR	Snake Pit	this study	no, too short	KX714874
S. pectinatus	PL5-SP-P72	MAR	Snake Pit	this study	yes	KX714892
S. pectinatus	PL5-SP-P73	MAR	Snake Pit	this study	yes	KX714891
S. pectinatus	PL5-SP-P74	MAR	Snake Pit	this study	yes	KX714902
S. pectinatus	PL12-TAG-P76	MAR	TAG	this study	yes	KX714890
S. pectinatus	PL12-TAG-P77	MAR	TAG	this study	yes	KX714889
S. pectinatus	PL12-TAG-P78	MAR	TAG	this study	yes	KX714875
S. pectinatus	PL12-TAG-P80	MAR	TAG	this study	yes	KX714888
S. pectinatus	PL12-TAG-P81	MAR	TAG	this study	yes	KX714887
S. pectinatus	PL12-TAG-P82	MAR	TAG	this study	yes	KX714886
S. pectinatus	PL12-TAG-P83	MAR	TAG	this study	yes	KX714885
S. pectinatus	PL12-TAG-P84	MAR	TAG	this study	yes	KX714884
S. pectinatus	PL12-TAG-P85	MAR	TAG	this study	yes	KX714903
S. pectinatus	PL12-TAG-P86	MAR	TAG	this study	yes	KX714904
S. pectinatus	PL12-TAG-P87	MAR	TAG	this study	yes	KX714883
S. pectinatus	PL12-TAG-P88	MAR	TAG	this study	no, too short	KX714871
S. pectinatus	PL12-TAG-P89	MAR	TAG	this study	yes	KX714882
S. pectinatus	PL12-TAG-P90	MAR	TAG	this study	yes	KX714881
S. pectinatus	PL12-TAG-P91	MAR	TAG	this study	yes	KX714880
S. pectinatus	PL12-TAG-P92	MAR	TAG	this study	yes	KX714905
S. pectinatus	PL12-TAG-P93	MAR	TAG	this study	yes	KX714879
S. pectinatus	PL12-TAG-P94	MAR	TAG	this study	yes	KX714878
S. pectinatus	PL12-TAG-P95	MAR	TAG	this study	yes	KX714906
S. pectinatus	P21	MAR	Turtle Pits Tower	Gollner et al. 2011	no, too many ambiguities	GQ925953

S. pectinatus	P20	MAR	Turtle Pits Tower	Gollner et al. 2011	no, too many ambiguities	GQ925952
S. hispidulus	S1291	EPR	Marker 28	Gollner et al. 2011	yes	GQ926174
S. hispidulus	S129	EPR	Marker 28	Gollner et al. 2011	yes	GQ926173
S. hispidulus	S128	EPR	Marker 28	Gollner et al. 2011	yes	GQ926172
S. hispidulus	S127	EPR	Marker 28	Gollner et al. 2011	yes	GQ926171
S. hispidulus	S126	EPR	Marker 28	Gollner et al. 2011	yes	GQ926170
S. hispidulus	S125	EPR	Marker 28	Gollner et al. 2011	yes	GQ926169
S. hispidulus	S123	EPR	Tica	Gollner et al. 2011	yes	GQ926168
S. hispidulus	S122	EPR	Tica	Gollner et al. 2011	yes	GQ926167
S. hispidulus	S121	EPR	Tica	Gollner et al. 2011	yes	GQ926166
S. hispidulus	S120	EPR	Marker 28	Gollner et al. 2011	yes	GQ926165
S. hispidulus	S119	EPR	Marker 28	Gollner et al. 2011	yes	GQ926164
S. hispidulus	S118	EPR	Bio 9	Gollner et al. 2011	yes	GQ926163
S. hispidulus	S117	EPR	Bio 9	Gollner et al. 2011	yes	GQ926162
S. hispidulus	S116	EPR	Bio 9	Gollner et al. 2011	yes	GQ926161
S. hispidulus	S115	EPR	Bio 9	Gollner et al. 2011	yes	GQ926160
S. hispidulus	S114	EPR	Bio 9	Gollner et al. 2011	yes	GQ926159
S. hispidulus	S113	EPR	Bio 9	Gollner et al. 2011	yes	GQ926158
S. hispidulus	S112	EPR	Bio 9	Gollner et al. 2011	no, too short	GQ926157
S. hispidulus	S111	EPR	Bio 9	Gollner et al. 2011	yes	GQ926156
S. hispidulus	S110	EPR	Bio 9	Gollner et al. 2011	yes	GQ926155
S. hispidulus	S109	EPR	Bio 9	Gollner et al. 2011	yes	GQ926154
S. hispidulus	S108	EPR	Bio 9	Gollner et al. 2011	yes	GQ926153
S. hispidulus	S107	EPR	Bio 9	Gollner et al. 2011	yes	GQ926152
S. hispidulus	S106	EPR	Bio 9	Gollner et al. 2011	yes	GQ926151
S. hispidulus	S105	EPR	Bio 9	Gollner et al. 2011	yes	GQ926150
S. hispidulus	S104	EPR	Bio 9	Gollner et al. 2011	yes	GQ926149
S. hispidulus	S103	EPR	Bio 9	Gollner et al. 2011	yes	GQ926148
S. hispidulus	S102	EPR	Bio 9	Gollner et al. 2011	yes	GQ926147
S. hispidulus	S101	EPR	Bio 9	Gollner et al. 2011	yes	GQ926146
S. hispidulus	S100	EPR	Bio 9	Gollner et al. 2011	yes	GQ926145
S. hispidulus	S99	EPR	Bio 9	Gollner et al. 2011	yes	GQ926144
S. hispidulus	S98	EPR	Bio 9	Gollner et al. 2011	yes	GQ926143
S. hispidulus	S97	EPR	Bio 9	Gollner et al. 2011	yes	GQ926142
S. hispidulus	S96	EPR	Bio 9	Gollner et al. 2011	yes	GQ926141
S. hispidulus	S95	EPR	Bio 9	Gollner et al. 2011	yes	GQ926140
S. hispidulus	S94	EPR	Bio 9	Gollner et al. 2011	yes	GQ926139
S. hispidulus	S93	EPR	Marker 28	Gollner et al. 2011	yes	GQ926138
S. hispidulus	S92	EPR	Marker 28	Gollner et al. 2011	yes	GQ926137
S. hispidulus	S91	EPR	Marker 28	Gollner et al. 2011	yes	GQ926136
S. hispidulus	S90	EPR	Marker 28	Gollner et al. 2011	yes	GQ926135
S. hispidulus	S89	EPR	Marker 28	Gollner et al. 2011	yes	GQ926134
S. hispidulus	S88	EPR	Marker 28	Gollner et al. 2011	yes	GQ926133

S. hispidulus	S86	EPR	Tica	Gollner et al. 2011	yes	GQ926132
S. hispidulus	S85	EPR	Tica	Gollner et al. 2011	yes	GQ926131
S. hispidulus	S83II	EPR	Tica	Gollner et al. 2011	yes	GQ926130
S. hispidulus	S82	EPR	Tica	Gollner et al. 2011	yes	GQ926129
S. hispidulus	S81	EPR	Bio 9	Gollner et al. 2011	yes	GQ926128
S. hispidulus	S80	EPR	Bio 9	Gollner et al. 2011	yes	GQ926127
S. hispidulus	S79	EPR	Bio 9	Gollner et al. 2011	yes	GQ926126
S. hispidulus	S78	EPR	Bio 9	Gollner et al. 2011	yes	GQ926125
S. hispidulus	S77	EPR	Bio 9	Gollner et al. 2011	yes	GQ926124
S. hispidulus	S76	EPR	Bio 9	Gollner et al. 2011	no, too short	GQ926123
S. hispidulus	S75	EPR	V-Vent	Gollner et al. 2011	yes	GQ926122
S. hispidulus	S74	EPR	V-Vent	Gollner et al. 2011	yes	GQ926121
S. hispidulus	S72II	EPR	V-Vent	Gollner et al. 2011	yes	GQ926119
S. hispidulus	S71	EPR	V-Vent	Gollner et al. 2011	yes	GQ926118
S. hispidulus	S69	EPR	Marker 28	Gollner et al. 2011	yes	GQ926117
S. hispidulus	S68	EPR	Marker 28	Gollner et al. 2011	yes	GQ926116
S. hispidulus	S67	EPR	V-Vent	Gollner et al. 2011	yes	GQ926115
S. hispidulus	S136	EPR	V-Vent	Gollner et al. 2011	yes	GQ926114
S. hispidulus	S135	EPR	V-Vent	Gollner et al. 2011	yes	GQ926113
S. hispidulus	S134II	EPR	Bio 9	Gollner et al. 2011	yes	GQ926112
S. hispidulus	S134	EPR	Bio 9	Gollner et al. 2011	yes	GQ926111
S. hispidulus	S133	EPR	V-Vent	Gollner et al. 2011	yes	GQ926110
S. hispidulus	S132II	EPR	V-Vent	Gollner et al. 2011	yes	GQ926109
S. hispidulus	S132	EPR	V-Vent	Gollner et al. 2011	yes	GQ926108
S. hispidulus	S131II	EPR	V-Vent	Gollner et al. 2011	yes	GQ926107
S. hispidulus	S131	EPR	V-Vent	Gollner et al. 2011	yes	GQ926106
S. hispidulus	S137	GC	Rebeccas Roost	Gollner et al. 2011	in overall calculation only	GQ925970
S. hispidulus	S52	EPR	Bio 9	Gollner et al. 2011	yes	GQ925969
S. hispidulus	S42M42	EPR	V-Vent	Gollner et al. 2011	yes	GQ925968
S. hispidulus	S23M42	EPR	V-Vent	Gollner et al. 2011	yes	GQ925967
S. hispidulus	S22M42	EPR	V-Vent	Gollner et al. 2011	yes	GQ925966
S. hispidulus	S21M42	EPR	V-Vent	Gollner et al. 2011	yes	GQ925965
S. hispidulus	S20M42	EPR	V-Vent	Gollner et al. 2011	yes	GQ925964
S. hispidulus	S19M42	EPR	V-Vent	Gollner et al. 2011	yes	GQ925963
S. hispidulus	S18M42	EPR	V-Vent	Gollner et al. 2011	yes	GQ925962
S. hispidulus	S13M42	EPR	V-Vent	Gollner et al. 2011	yes	GQ925961
S. hispidulus	S12M42	EPR	V-Vent	Gollner et al. 2011	yes	GQ925960
S. hispidulus	S7M42	EPR	V-Vent	Gollner et al. 2011	yes	GQ925959
S. hispidulus	S1	EPR	V-Vent	Gollner et al. 2011	yes	GQ925958
S. hispidulus	S2	EPR	V-Vent	Gollner et al. 2011	yes	GQ925957
S. hispidulus	S3	EPR	V-Vent	Gollner et al. 2011	yes	GQ925956
S. hispidulus	S4	EPR	V-Vent	Gollner et al. 2011	yes	GQ925955
S. hispidulus	S5	EPR	V-Vent	Gollner et al. 2011	yes	GQ925954

S. lauensis	L98	ELSC	Kilo Moana		yes	KX714907
S. lauensis	L99	ELSC	Kilo Moana		yes	KX714908
S. lauensis	L79	ELSC	ABE	Gollner et al. 2011	yes	GQ926105
S. lauensis	L78	ELSC	ABE	Gollner et al. 2011	yes	GQ926104
S. lauensis	L77	ELSC	ABE	Gollner et al. 2011	yes	GQ926103
S. lauensis	L76	ELSC	ABE	Gollner et al. 2011	yes	GQ926102
S. lauensis	L75	ELSC	ABE	Gollner et al. 2011	yes	GQ926101
S. lauensis	L74	ELSC	ABE	Gollner et al. 2011	yes	GQ926100
S. lauensis	L73	ELSC	Kilo Moana	Gollner et al. 2011	yes	GQ926099
S. lauensis	L72	ELSC	Kilo Moana	Gollner et al. 2011	yes	GQ926098
S. lauensis	L71	ELSC	Kilo Moana	Gollner et al. 2011	yes	GQ926097
S. lauensis	L70	ELSC	Kilo Moana	Gollner et al. 2011	yes	GQ926096
S. lauensis	L69	ELSC	Kilo Moana	Gollner et al. 2011	yes	GQ926095
S. lauensis	L68	ELSC	Tu'i Malila	Gollner et al. 2011	yes	GQ926094
S. lauensis	L67	ELSC	Tu'i Malila	Gollner et al. 2011	yes	GQ926093
S. lauensis	L66	ELSC	Tu'i Malila	Gollner et al. 2011	yes	GQ926092
S. lauensis	L64	ELSC	ABE	Gollner et al. 2011	yes	GQ926091
S. lauensis	L63	ELSC	ABE	Gollner et al. 2011	yes	GQ926090
S. lauensis	L62	ELSC	ABE	Gollner et al. 2011	yes	GQ926089
S. lauensis	L61	ELSC	ABE	Gollner et al. 2011	yes	GQ926088
S. lauensis	L60	ELSC	ABE	Gollner et al. 2011	yes	GQ926087
S. lauensis	L59	ELSC	ABE	Gollner et al. 2011	yes	GQ926086
S. lauensis	L58	ELSC	ABE	Gollner et al. 2011	yes	GQ926085
S. lauensis	L57	ELSC	ABE	Gollner et al. 2011	yes	GQ926084
S. lauensis	L56	ELSC	ABE	Gollner et al. 2011	yes	GQ926083
S. lauensis	L55	ELSC	ABE	Gollner et al. 2011	yes	GQ926082
S. lauensis	L54	ELSC	ABE	Gollner et al. 2011	yes	GQ926081
S. lauensis	L53	ELSC	Tu'i Malila	Gollner et al. 2011	yes	GQ926080
S. lauensis	L52	ELSC	Tu'i Malila	Gollner et al. 2011	yes	GQ926079
S. lauensis	L51	ELSC	Tu'i Malila	Gollner et al. 2011	yes	GQ926078
S. lauensis	L50	ELSC	Tu'i Malila	Gollner et al. 2011	yes	GQ926077
S. lauensis	L49	ELSC	Tu'i Malila	Gollner et al. 2011	yes	GQ926076
S. lauensis	L48	ELSC	Tu'i Malila	Gollner et al. 2011	yes	GQ926075
S. lauensis	L47	ELSC	Tu'i Malila	Gollner et al. 2011	yes	GQ926074
S. lauensis	L46	ELSC	Tu'i Malila	Gollner et al. 2011	yes	GQ926073
S. lauensis	L45	ELSC	Tu'i Malila	Gollner et al. 2011	yes	GQ926072
S. lauensis	L44	ELSC	Tu'i Malila	Gollner et al. 2011	yes	GQ926071
S. lauensis	L43	ELSC	Tu'i Malila	Gollner et al. 2011	yes	GQ926070
S. lauensis	L42	ELSC	Tu'i Malila	Gollner et al. 2011	yes	GQ926069
S. lauensis	L41	ELSC	Tu'i Malila	Gollner et al. 2011	yes	GQ926068
S. lauensis	L40	ELSC	Tu'i Malila	Gollner et al. 2011	yes	GQ926067
S. lauensis	L39	ELSC	Tu'i Malila	Gollner et al. 2011	yes	GQ926066
S. lauensis	L38	ELSC	Tu'i Malila	Gollner et al. 2011	yes	GQ926065
S. lauensis	L37	ELSC	Tu'i Malila	Gollner et al. 2011	yes	GQ926064

S. lauensis	L36	ELSC	Tu'i Malila	Gollner et al. 2011	yes	GQ926063
S. lauensis	L35	ELSC	Tu'i Malila	Gollner et al. 2011	yes	GQ926062
S. lauensis	L34	ELSC	Tu'i Malila	Gollner et al. 2011	yes	GQ926061
S. lauensis	L33	ELSC	ABE	Gollner et al. 2011	yes	GQ926060
S. lauensis	L32	ELSC	ABE	Gollner et al. 2011	yes	GQ926059
S. lauensis	L31	ELSC	ABE	Gollner et al. 2011	yes	GQ926058
S. lauensis	L30	ELSC	ABE	Gollner et al. 2011	yes	GQ926057
S. lauensis	L29	ELSC	ABE	Gollner et al. 2011	yes	GQ926056
S. lauensis	L28	ELSC	ABE	Gollner et al. 2011	yes	GQ926055
S. lauensis	L27	ELSC	ABE	Gollner et al. 2011	yes	GQ926054
S. lauensis	L26	ELSC	ABE	Gollner et al. 2011	yes	GQ926053
S. lauensis	L25	ELSC	ABE	Gollner et al. 2011	yes	GQ926052
S. lauensis	L24	ELSC	ABE	Gollner et al. 2011	yes	GQ926051
S. lauensis	L23	ELSC	ABE	Gollner et al. 2011	yes	GQ926050
S. lauensis	L22	ELSC	ABE	Gollner et al. 2011	yes	GQ926049
S. lauensis	L21	ELSC	ABE	Gollner et al. 2011	yes	GQ926048
S. lauensis	L20	ELSC	ABE	Gollner et al. 2011	yes	GQ926047
S. lauensis	L19	ELSC	ABE	Gollner et al. 2011	yes	GQ926046
S. lauensis	L18	ELSC	ABE	Gollner et al. 2011	yes	GQ926045
S. lauensis	L17	ELSC	ABE	Gollner et al. 2011	yes	GQ926044
S. lauensis	L16	ELSC	ABE	Gollner et al. 2011	yes	GQ926043
S. lauensis	L15	ELSC	ABE	Gollner et al. 2011	yes	GQ926042
S. lauensis	L14	ELSC	ABE	Gollner et al. 2011	yes	GQ926041
S. lauensis	L13	ELSC	ABE	Gollner et al. 2011	yes	GQ926040
S. lauensis	L12	ELSC	ABE	Gollner et al. 2011	yes	GQ926039
S. lauensis	L11	ELSC	ABE	Gollner et al. 2011	yes	GQ926038
S. lauensis	L10	ELSC	ABE	Gollner et al. 2011	yes	GQ926037
S. lauensis	L9	ELSC	ABE	Gollner et al. 2011	yes	GQ926036
S. lauensis	L8	ELSC	ABE	Gollner et al. 2011	yes	GQ926035
S. lauensis	L7	ELSC	ABE	Gollner et al. 2011	yes	GQ926034
S. lauensis	L6	ELSC	ABE	Gollner et al. 2011	yes	GQ926033
S. lauensis	L5	ELSC	ABE	Gollner et al. 2011	yes	GQ926032
S. lauensis	L4	ELSC	ABE	Gollner et al. 2011	yes	GQ926031
S. lauensis	L3	ELSC	ABE	Gollner et al. 2011	yes	GQ926030
S. lauensis	L2	ELSC	ABE	Gollner et al. 2011	yes	GQ926029
S. lauensis	L1	ELSC	ABE	Gollner et al. 2011	yes	GQ926028
S. brevispina	B28	ELSC	ABE	Gollner et al. 2011	in overall calculation only	GQ926007
S. brevispina	B27E	ELSC	Tu'i Malila	Gollner et al. 2011	yes	GQ926006
S. brevispina	B27	ELSC	Tu'i Malila	Gollner et al. 2011	yes	GQ926005
S. brevispina	B26E	ELSC	Tu'i Malila	Gollner et al. 2011	yes	GQ926004
S. brevispina	B26	ELSC	Tu'i Malila	Gollner et al. 2011	yes	GQ926003
S. brevispina	B25E2	ELSC	Tu'i Malila	Gollner et al. 2011	yes	GQ926002
S. brevispina	B25E1	ELSC	Tu'i Malila	Gollner et al. 2011	yes	GQ926001

S. brevispina	B25	ELSC	Tu'i Malila	Gollner et al. 2011	yes	GQ926000
S. brevispina	B24E	ELSC	Tu'i Malila	Gollner et al. 2011	yes	GQ925999
S. brevispina	B24	ELSC	Tu'i Malila	Gollner et al. 2011	yes	GQ925998
S. brevispina	B23	ELSC	Tu'i Malila	Gollner et al. 2011	yes	GQ925997
S. brevispina	B22	ELSC	Tu'i Malila	Gollner et al. 2011	yes	GQ925996
S. brevispina	B21	ELSC	Tu'i Malila	Gollner et al. 2011	yes	GQ925995
S. brevispina	B19	ELSC	Tu'i Malila	Gollner et al. 2011	yes	GQ925994
S. brevispina	B18	ELSC	Tu'i Malila	Gollner et al. 2011	yes	GQ925993
S. brevispina	B17	ELSC	Tu'i Malila	Gollner et al. 2011	yes	GQ925992
S. brevispina	B16	ELSC	Tu'i Malila	Gollner et al. 2011	yes	GQ925991
S. brevispina	B15	ELSC	Tu'i Malila	Gollner et al. 2011	yes	GQ925990
S. brevispina	B14	ELSC	Tu'i Malila	Gollner et al. 2011	yes	GQ925989
S. brevispina	B13	ELSC	Tu'i Malila	Gollner et al. 2011	yes	GQ925988
S. brevispina	B12	ELSC	Tu'i Malila	Gollner et al. 2011	yes	GQ925987
S. brevispina	B11	ELSC	Tu'i Malila	Gollner et al. 2011	yes	GQ925986
S. brevispina	B10	ELSC	Tu'i Malila	Gollner et al. 2011	yes	GQ925985
S. brevispina	B9E	ELSC	Tu'i Malila	Gollner et al. 2011	no, too short; re-identified as S. lauensis egg	GQ925984
S. brevispina	B9	ELSC	Tu'i Malila	Gollner et al. 2011	yes	GQ925983
S. brevispina	B8E	ELSC	Tu'i Malila	Gollner et al. 2011	yes	GQ925982
S. brevispina	B8	ELSC	Tu'i Malila	Gollner et al. 2011	yes	GQ925981
S. brevispina	B7	ELSC	Tu'i Malila	Gollner et al. 2011	yes	GQ925980
S. brevispina	B6	ELSC	Kilo Moana	Gollner et al. 2011	yes	GQ925979
S. brevispina	B5	ELSC	Kilo Moana	Gollner et al. 2011	yes	GQ925978
S. brevispina	B4	ELSC	Kilo Moana	Gollner et al. 2011	yes	GQ925977
S. brevispina	B3E	ELSC	Kilo Moana	Gollner et al. 2011	yes	GQ925976
S. brevispina	B3	ELSC	Kilo Moana	Gollner et al. 2011	yes	GQ925975
S. brevispina	B2	ELSC	Kilo Moana	Gollner et al. 2011	yes	GQ925974
S. brevispina	B1E	ELSC	Kilo Moana	Gollner et al. 2011	yes	GQ925973
S. brevispina	B1	ELSC	Kilo Moana	Gollner et al. 2011	yes	GQ925972
A. limatulus	AL1	EPR	Eastwall	this study	yes	KX714845
A. limatulus	AL2	EPR	Eastwall	this study	yes	KX714846
A. limatulus	AL3	EPR	Eastwall	this study	yes	KX714847
A. limatulus	AL4	EPR	Eastwall	this study	yes	KX714848
A. limatulus	AL5	EPR	P-Vent	this study	yes	KX714849
A. mammillatus	A16	EPR	Tica	this study	yes	KX714835
A. mammillatus	A17	EPR	Tica	this study	yes	KX714832
A. mammillatus	A20	EPR	Tica	this study	yes	KX714836
A. mammillatus	A21	EPR	Tica	this study	yes	KX714831
A. mammillatus	A22	EPR	Tica	this study	yes	KX714837
A. mammillatus	A33	EPR	Tica	this study	yes	KX714838
A. mammillatus	A34	EPR	Tica	this study	no, too short	KX714834
A. mammillatus	A36	EPR	Tica	this study	yes	KX714830

A. mammillatus	A37	EPR	Tica	this study	yes	KX714839
A. mammillatus	A38	EPR	Tica	this study	yes	KX714840
A. mammillatus	A40	EPR	Tica	this study	yes	KX714833
A. mammillatus	A41	EPR	Eastwall	this study	yes	KX714841
A. mammillatus	A42	EPR	Eastwall	this study	yes	KX714842
A. mammillatus	A43	EPR	Eastwall	this study	yes	KX714843
A. mammillatus	A44	EPR	Eastwall	this study	yes	KX714844
Ameira sp. nov.	AM27	EPR	Eastwall	this study	no	KX714911
Ameira sp. nov.	AM18	EPR	Sketchy	this study	no	KX714912
Amphiascus sp. nov.	AMP7	ELSC	ABE	this study	no	KX714909
Amphiascus sp. nov.	AMPE6	EPR	Tica	this study	no	KX714910
