# Supplementary material for: Mitochondrial DNA Analyses Indicate High Diversity, Expansive Population Growth and High Genetic Connectivity of Vent Copepods (Dirivultidae) across Different Oceans
Source: PLoS One. 2016 Oct 12;11(10):e0163776. doi: 10.1371/journal.pone.0163776 (PMC5061364; doi:10.1371/journal.pone.0163776)
Supplement: S2 Table — Identification number of simulation (id), divergence time in generations (tau), mutation and recombination rates of 1x10^-8^ events/per bp/ per generation (m), number of individuals per population (Ne), number of migrants per generation (migr./gen.), length of loci in base pairs (bp), mjgration type, number of pseudo observed data (pseu.obs.), and number of replicates per pseudo observed sample (repl.pseu.obs.). (PDF) [file pone.0163776.s003.pdf]

S2 Table. **Simulated datasets that were used as input to the maximum-likelihood parameter estimation procedure implemented in *fastsimcoal2.*** Identification number of simulation (id), divergence time in generations (tau), mutation and recombination rates of $1x10^{-8}$ events/per bp/ per generation (m), number of individuals per population (Ne), number of migrants per generation (migr./gen.), length of loci in base pairs (bp), mjgration type, number of pseudo observed data (pseu.obs.), and number of replicates per pseudo observed sample (repl.pseu.obs.)

id	T_{DIV}	m	Ne	migr./gen.	Number of fragments	Fragment length	migration type	Simulated datasets	Re-estimations per simulated dataset
1	100000	0.0001	10000	1	10	600 bp	symetrical	20	20
2	100000	0.0001	10000	1	100	600 bp	symetrical	20	20
3	100000	0.0001	10000	1	1000	600 bp	symetrical	20	20
4	100000	0.0001	10000	1	10000	600 bp	symetrical	20	20
5	100000	0.0001	10000	1	10	600 bp	asymetrical	20	20
6	100000	0.0001	10000	1	100	600 bp	asymetrical	20	20
7	100000	0.0001	10000	1	1000	600 bp	asymetrical	20	20
8	100000	0.0001	10000	1	10000	600 bp	asymetrical	20	20
9	1000	0.0001	10000	1	10	600 bp	symetrical	20	20
10	1000	0.0001	10000	1	100	600 bp	symetrical	20	20
11	1000	0.0001	10000	1	1000	600 bp	symetrical	20	20
12	1000	0.0001	10000	1	10000	600 bp	symetrical	20	20
13	1000	0.0001	10000	1	10	600 bp	asymetrical	20	20
14	1000	0.0001	10000	1	100	600 bp	asymetrical	20	20
15	1000	0.0001	10000	1	1000	600 bp	asymetrical	20	20
16	1000	0.0001	10000	1	10000	600 bp	asymetrical	20	20
