# Supplementary material for: Mitochondrial DNA Analyses Indicate High Diversity, Expansive Population Growth and High Genetic Connectivity of Vent Copepods (Dirivultidae) across Different Oceans
Source: PLoS One. 2016 Oct 12;11(10):e0163776. doi: 10.1371/journal.pone.0163776 (PMC5061364; doi:10.1371/journal.pone.0163776)
Supplement: S3 Table — (PDF) [file pone.0163776.s004.pdf]

S3 Table. **Average p-distance between taxa (below diagonal) and respective standard error calculations based on 500 bootstrap replicates (above diagonal).**

Taxon	1	2	3	4	5	6	7	8	9	10
1 Stygiopontius sp.1		0.017	0.0178	0.0149	0.0156	0.0163	0.016	0.0171	0.0168	0.0182
2 Stygiopontius sp.2	0.2184		0.0168	0.0171	0.0171	0.0175	0.0177	0.0181	0.0181	0.0166
3 Stygiopontius pectinatus	0.2345	0.2049		0.0183	0.0191	0.0185	0.0173	0.0177	0.018	0.0171
4 Stygiopontius hispidulus	0.1784	0.2489	0.2822		0.0155	0.0151	0.0164	0.0161	0.0164	0.0162
5 Stygiopontius lauensis	0.1892	0.2282	0.2618	0.2034		0.0135	0.0161	0.016	0.0161	0.0174
6 Stygiopontius brevispina	0.1988	0.2486	0.2619	0.2236	0.1461		0.0163	0.0166	0.0165	0.0167
7 Aphotopontius sp.1	0.2179	0.2725	0.2974	0.2348	0.2558	0.2483		0.0176	0.0165	0.0172
8 Aphotopontius limatulus	0.2375	0.2956	0.2992	0.2654	0.2459	0.2587	0.2651		0.0162	0.0177
9 Aphotopontius mammillatus	0.2727	0.2931	0.3101	0.2877	0.2755	0.2974	0.254	0.2311		0.0171
10 Chasmatopontius sp.1	0.2518	0.2333	0.261	0.2529	0.2679	0.2642	0.2793	0.3096	0.3078	
