# Supplementary material for: Mitochondrial DNA Analyses Indicate High Diversity, Expansive Population Growth and High Genetic Connectivity of Vent Copepods (Dirivultidae) across Different Oceans
Source: PLoS One. 2016 Oct 12;11(10):e0163776. doi: 10.1371/journal.pone.0163776 (PMC5061364; doi:10.1371/journal.pone.0163776)
Supplement: S4 Table — Site, year of collection, number of samples (n), and species are given. Abundance data from sites Sketchy, P-Vent and Tica were extracted from raw data from Gollner et al. 2015. Data from sites Bio9 and Eastwall are here published for the first time. Information on sampling strategies and methods in Gollner et al. 2013 and Gollner et al. 2015. (PDF) [file pone.0163776.s005.pdf]

S4 Table. **Mean copepod abundance per 64 cm^2 in artificial settlement devices used to study recovery of fauna on the 9°N East Pacific Rise after the 2006 eruption.** Site, year of collection, number of samples (n), and species are given. Abundance data from sites Sketchy, P-Vent and Tica were extracted from raw data from Gollner et al. 2015. Data from sites Bio9 and Eastwall are here published for the first time. Information on sampling strategies and methods in Gollner et al. 2013 and Gollner et al. 2015 (1, 2).

Site and year collected	n	*A. limatulus*	*A. mammillatus*	*S. hispidulus*
Sketchy 2006	3	0.3 ± 0.8	0.3 ± 0.3	0
P-Vent 2006	3	0	0	0.5 ± 0.9
P-Vent 2007	3	0	0	0
Tica 2006	3	0.2 ± 1	0	1 ± 1.4
Tica2007	3	0.2 ± 0.3	0	0
Tica 2009	3	0.5 ± 0.9	0.2 ± 0.3	0
Bio9 2006	1	0	0	239
Eastwall 2006	3	0.1 ± 0.1	0.1 ± 0.1	4.1 ± 3.6
Eastwall 2007	2	0	0	0
Eastwall 2009	2	66.2 ± 81.6	8.4 ± 11.9	0

1. Gollner, S., Miljutina, M., Bright, M. Nematode succession at deep-sea hydrothermal vents after a recent volcanic eruption with the desciption of two dominant species. Org Div Evol. 2013;13:349-71.
2. Gollner, S., Govenar, B., Martinez Arbizu, P., Mills, S., Le Bris, N., Weinbauer, M., Shank, T.M., Bright, M. Differences in recovery between deep-sea hydrothermal vent and vent-proximate communities after a volcanic eruption. Deep-Sea Res I. 2015;106:167-82.
